# Development and validation of the UHPLC-MS/MS method for the quantitative determination of 25 PFAS in dried blood spots

**DOI:** 10.1007/s00216-024-05484-6

**Published:** 2024-08-19

**Authors:** Martina Galletto, Christina Ververi, Marta Massano, Eugenio Alladio, Marco Vincenti, Alberto Salomone

**Affiliations:** 1 Department of Chemistry, University of Turin, Turin, Italy; 2 Centro Regionale Antidoping, Orbassano, TO Italy

**Keywords:** PFAS, DBS, UHPLC-MS/MS, Occupational exposure, Health monitoring

## Abstract

Per- and polyfluoroalkyl substances (PFAS) are anthropogenic fluorine-containing compounds largely used in industrial and consumer applications. They tend to bioaccumulate in the human body after intake from various sources in daily life. Following repeated exposure to PFAS, a broad range of adverse health outcomes has been reported. Consequently, monitoring PFAS levels in human blood is of paramount importance for public health policies. In contrast with traditional venipuncture, dried blood spots (DBS) constitute a reliable, cheap, and less invasive technique to allow microsampling by capillary blood collected on a specific device. This work aimed to develop and validate an innovative analytical method, combining quantitative DBS with UHPLC-MS/MS instrumentation to identify and quantify 25 PFAS. The extraction procedure was developed and optimized within the range 2–100 ng/mL. Specifically, fortified blood was applied on *Capitainer*®*B* devices providing 10 μL of blood volume through a microfluidic channel. After 3 h of drying, the extraction was performed by methanol under sonication, followed by centrifugation. Then, the extraction solvent was evaporated; the residue was reconstituted with the mobile phase solution. The validated method evidenced good sensitivity, with limits of detection ranging from 0.4 ng/mL (PFODA, PFOS) to 1.0 ng/mL (PFOA, 3,6-OPFHpA). The ± 20% acceptability criteria established for intra- and inter-day precision and accuracy were fulfilled for all analytes. High recovery—above 80%—was recorded, whereas significant matrix effect resulted in ion enhancement (> 50%) for 13 analytes. In conclusion, the proposed workflow proved to be reliable, fit for purpose, and easily adaptable in the laboratory routine.

**Graphical Abstract:**

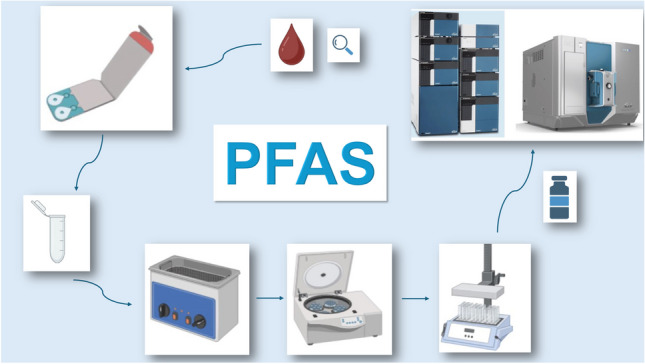

**Supplementary Information:**

The online version contains supplementary material available at 10.1007/s00216-024-05484-6.

## Introduction

Per- and polyfluoroalkyl substances, known as PFAS, represent a class of synthetically produced chemicals. Generally, they contain at least one perfluoroalkyl moiety in which all hydrogen atoms bonded to the aliphatic carbon atoms have been replaced by fluorine atoms. A polar functional group, either a carboxylic or sulfonic acid, is commonly present at the end of the alkyl chain [1, 2]. Actually, the most abundant PFAS subgroups identified in environmental and biological matrices are perfluorocarboxylic acids (PFCAs) and perfluoroalkylsulfonic acids (PFSAs). Another significant sub-group of PFAS includes an ether bond [3]. The extraordinary strength of the C-F bond provides unique properties, offering a wide variety of industrial, commercial, domestic, and consumer applications since the 1940s [4].

PFAS are also colloquially known as “forever chemicals,” highlighting their high stability even in hostile conditions and their durable resistance to degradation under different conditions and matrices, including heat, water, and oil [5]. These features offer countless benefits for the production requirements in aerospace, military, automotive, textile, electronic, and construction fields [6].

However, the aforementioned quality comes with noticeable long-term drawbacks: PFAS are ubiquitous in the environment like persistent organic pollutants (POPs), and tend to bioaccumulate in tissues and fluids of human and animal bodies [7, 8]. Regarding human exposure to PFAS, it is crucial to evaluate the potential harm that may occur from relevant environmental or occupational exposure [9, 10]. Extensive studies showed a broad range of adverse health effects depending on the exposure conditions and other factors related to age, gender, ethnicity, and genetic predisposition [7]. Humans may incorporate PFAS by dermal absorption, inhalation, ingestion of potable water, and contaminated food [11]. Toxicological effects reported in the literature comprise immunosuppression, thyroid hormone disruption, carcinogenicity connected to kidney and gonads, liver disease, and lipid profile alteration [7, 12]. Furthermore, it has been shown that PFAS can both cross the placenta and be transferred to the newborn during breastfeeding, possibly causing neurodevelopmental deficiencies and developmental delay consequent to intrauterine growth restriction [13, 14]. In light of the persistence of these compounds and their hazard for human and animals, several analytical methods are regularly developed for their detection in various biological and environmental matrices [15, 16].

In this context, human biomonitoring (HBI) plays a key role in providing information about individual exposure levels, even if HBI studies are unable to predict precisely the adverse effects [17, 18]. Previous research established that moderate adverse outcomes may potentially occur, especially in sensitive life stages, when the sum of PFAS concentrations in serum or plasma reach a level ranging between 2 and 20 ng/mL, while further increased health risks occur if it exceeds 20 ng/mL [19].

The matrix of choice for PFAS determination is blood, because of the strong PFAS binding to serum albumin [20] and long half-lives (PFOA 3.8 years; PFOS 5.0 years) [7, 21]. Various analytical methods using plasma or serum have been developed in the last years, taking advantage of favorable partitioning in this biological fluid fraction [22, 23]. In contrast, whole blood has been rarely used in toxicological and clinical PFAS determinations because of the difficulties related to storage conditions and analytes’ stability [20].

A valid alternative to intravenous blood sampling is offered by dried blood spots (DBS), a microsampling technique based on a capillary blood drop—typically obtained by finger pricking—applied on a specially manufactured filter paper and then dried [24]. The use of DBS presents several advantages over intravenous blood collection, including reduced discomfort for the tested subjects, lower shipping costs and storage requirements, enhanced stability, and smaller biological specimens [25, 26]. Moreover, DBS is a more sustainable technique as it complies with the “3Rs” principles (reduction, refinement, and replacement) [27]. Nowadays, several microsampling technologies are available and employed for biomedical applications [27, 28]. Among them, devices consisting of microfluidic channels are increasingly applied in analytical methods, thus ensuring accurate volumetric sampling, consequently avoiding the variability associated to hematocrit values occurring when a traditional non-volumetric filter paper is used [29].

The present study aimed to develop and validate a quantitative analytical method addressed to a selected panel of 25 PFAS present in volumetric DBS devices, by means of ultra-high-performance liquid chromatography coupled to tandem mass spectrometry (UHPLC-MS/MS). The main forthcoming application is to monitor PFAS levels in individuals who may be exposed, either because of their occupation or residence in contaminated areas.

## Materials and methods

### Reagents and standards

Methanol, acetonitrile, and ammonium formate (for HPLC, purity ≥ 99%) were purchased from Sigma-Aldrich (Milan, Italy). Ultra-pure water was obtained from a Milli-Q® UF-Plus apparatus (Millipore, Bedford, MA, USA). 25 PFAS compounds and 9 mass-labelled ^13^C internal standards were purchased from Wellington Laboratories (Guelph, Ontario, Canada). Following the comprehensive list: perfluoro-n-butanoic acid (PFBA); perfluoro-n-pentanoic acid (PFPeA); perfluoro-n-hexanoic acid (PFHxA); perfluoro-n-heptanoic acid (PFHpA); perfluoro-n-octanoic acid (PFOA); perfluoro-n-nonanoic acid (PFNA); perfluoro-n-decanoic acid (PFDA); perfluoro-n-undecanoic acid (PFUdA); perfluoro-n-dodecanoic acid (PFDoA); perfluoro-n-tridecanoic acid (PFTrDA); perfluoro-n-tetradecanoic acid (PFTeDA); perfluoro-n-hexadecanoic acid (PFHxDA); perfluoro-n-octadecanoic acid (PFODA); perfluoro-1-butanesulfonic acid (L-PFBS); perfluoro-1-hexanesulfonic acid (L-PFHxS); perfluoro-1-octanesulfonic acid (L-PFOS); perfluoro-1-decanesulfonic acid (L-PFDS); 2,3,3,3-tetrafluoro-2-(1,1,2,2,3,3,3-heptafluoroproproxy) propanoic acid (HFPO-DA); dodecafluoro-3H-4,8-dioxanonanoic acid (ADONA); 9-chlorohexadecafluoro-3-oxanonane-1-sulfonic acid (9Cl-PF3ONS); 11-chloroeicosafluoro-3-oxaundecane-1-sulfonic acid (11Cl-PF3OUdS); perfluoro-4-oxapentanoic acid (PF4OPeA); perfluoro-5-oxahexanoic acid (PF5OHxA); perfluoro (2-ethoxyethane)sulfonic acid; perfluoro-3,6-dioxaheptanoic acid (3,6-OPFHpA); perfluoro-n-(^13^C_4_) butanoic acid (MPFBA); perfluoro-n-(1,2-^13^C_2_) hexanoic acid (MPFHxA); perfluoro-n-(1,2,3,4-^13^C_4_) octanoic acid (MPFOA); perfluoro-n-(1,2,3,4,5-^13^C_5_) nonanoic acid (MPFNA); perfluoro-n-(1,2-^13^C_2_) decanoic acid (MPFDA); perfluoro-n-(1,2-^13^C_2_) undecanoic acid (MPFUdA); perfluoro-n-(1,2-^13^C_2_) dodecanoic acid (MPFDoA); perfluoro-1-hexane (^18^O_2_) sulfonic acid (MPFHxS); perfluoro-1-(1,2,3,4-^13^C_4_) octanesulfonic acid (MPFOS).

Stock solutions were used at a concentration of 2 μg/mL. Working solutions were prepared at the final concentrations of 500 ng/mL and 100 ng/mL by dilution with methanol. The internal standard working solution was prepared at a final concentration of 50 ng/mL. Reference materials were stored in amber glass vials at − 20 °C, according to the supplier’s recommendations until their use.

Microsampling devices used for the method development and validation were *Capitainer*®*B* cards.

### Sample preparation

A pooled whole blood was created by mixing equal parts of venous blood collected from three non-exposed volunteers in EDTA Vacutainer tubes and stored at 4 °C. The volunteers were recruited at the University of Turin, Italy.

Prior to that, each whole blood sample and the pool were tested for PFAS and declared suited for functioning as blank samples, since their PFAS levels were homogeneously below the method’s quantification limits.

Blood aliquots of 100 μL were pipetted into 2 mL polypropylene Eppendorf vials and fortified with the working solutions at six concentration levels (2–5-10–20-50–100 ng/mL). Fifty microliters of fortified blood was deposited into the blood inlet of a *Capitainer*®*B* card to ensure a smooth flow inside the microfluidic channel; by this system, a quantitative spot of 10 μL of blood is generated on the sample disc, while the excess is collected in a separated disc.

The spots were then allowed to dry for 3 h in the dark, at room temperature and controlled humidity. Dried spots were peeled off using a *Capitainer*®*B* tweezer and placed into a 2 mL polypropylene Eppendorf, in which the bottom diameter was larger than 6 mm (spot diameter).

Five hundred microliters of methanol was added as the extraction solvent together with 4 μL of stable isotope-labelled internal standard at 50 ng/mL (20 ng/mL is the final concentration in the DBS sample of 10 μL). After vortex mixing for 10 s, extraction was carried out by sonicating for 30 min at room temperature. Then, the sample was centrifuged for 10 min at 4000 rpm. After the spot removal from the Eppendorf, the extraction solvent was evaporated under a gentle nitrogen flow at room temperature and reconstituted with 20 μL of a 75:25 aqueous:organic mobile phase solution. Once transferred into the injection vials, samples were centrifuged again at 4000 rpm for 5 min and finally 3 μL solution was injected into the UHPLC-MS/MS.

### Quality controls and procedural blanks

Quality controls (QC) were prepared in three replicates for each level at low (2 ng/mL), medium (15 ng/mL), and high (75 ng/mL) concentrations using the same procedure described for calibrators. QCs were injected for every analytical session to monitor the instrumental reproducibility [29].

Additionally, different procedural blanks were processed to assess the potential background contamination. To do so, the same extraction protocol was applied to (1) dried blood on the spot without analytical standards; (2) sample disc; (3) 500 μL of extraction solvent pipetted into the Eppendorf; (4) injection vial cap; and (5) the mobile phase solution used to reconstitute samples.

### Instrumentation

PFAS analysis was performed on a UHPLC SCIEX ExionLC™ (AB SCIEX, Framingham, USA) coupled with a SCIEX 7500 TripleQuad (Darmstadt, Germany) equipped with an electrospray ion source (ESI). The SCIEX OS software was used for qualitative and quantitative data processing.

The UHPLC system was equipped with a C18 Luna Omega column (100 × 2.1 mm, 1.6 μm), by Phenomenex (Torrance, CA, USA), maintained at the temperature of 45 °C during the whole elution. To delay possible pefluorinated contaminants originating from the LC equipment, a Phenomenex XB-C18 Kinetex column (50 × 2.1 mm, 2.6 μm) was installed between the pump exit and the injector valve. The mobile phase was made of 2 mM ammonium formate in water as eluent A and an 80:20 v/v methanol/acetonitrile solution as eluent B. The flow rate was set at 0.5 mL/min. The gradient was as follows (A:B, v-v): isocratic elution 75:25 for 0.5 min, linear elution to 5:95 for 9.5 min, isocratic elution for 2 min, and equilibration at initial condition for 1 min, resulting in a 13-min total run time.

The ESI operated in negative ion mode. The source parameters were set as follows: temperature at 400 °C, spray voltage at 1500 V, ion source gas 1 at 30 psi, ion source gas 2 at 70 psi, curtain gas at 40 psi, collision gas (CAD) at 7 psi.

The targeted analysis was based on selected reaction monitoring (SRM) using two MS/MS transitions at the expected retention time to identify each molecule, with the exception of PFBA and PFPeA, for which only one transition was available.

The entrance potential (EP) was fixed at − 8 V for all analytes, while appropriate collision energies (CE) and collision cell exit potential (CXP) were adjusted for each ion. Different dwell times were tested to optimize the good peak shape yielding a final time of 10 ms. Retention times, precursor and product ion *m/z* values, and collision parameters for the 25 analytes and 9 internal standards are listed in Table 1.

**Table 1:** Retention time, precursor ion, product ions and optimized collision parameters for the 25 selected PFAS and the 9 internal standards. The first product ion in the list has been used for quantification

Analyte	Retention time (min)	Precursor ion mass Q1 (*m/z*)	Product ion mass Q3 (*m/z*)	CE (V)	CXP (V)
Perfluorocarboxylic acids					
PFBA	1.9	213.0	169.0	− 15	− 14
PFPeA	3.5	263.0	219.0	− 11	− 14
PFHxA	4.9	313.0	269.0 119.0	− 12 − 26	− 5 − 11
PFHpA	5.9	363.0	319.0 169.0	− 12 − 16	− 5 − 7
PFOA	6.7	413.0	369.0 169.0	− 14 − 18	− 7 − 7
PFNA	7.3	463.0	419.0 219.0	− 14 − 18	− 7 − 7
PFDA	7.8	513.0	469.0 219.0	− 14 − 18	− 7 − 7
PFUdA	8.2	563.0	519.0 269.0	− 18 − 18	− 7 − 7
PFDoA	8.7	613.0	569.0 169.0	− 18 − 26	− 7 − 13
PFTrDA	9.0	663.0	619.0 169.0	− 20 − 35	− 10 − 10
PFTeDA	9.3	713.0	669.0 169.0	− 22 − 35	− 10 − 10
PFHxDA	9.8	813.0	769.0 169.0	− 24 − 32	− 10 − 11
PFODA	10.2	913.0	869.0 169.0	− 26 − 36	− 11 − 15
Perfluorosulfonic acids					
L-PFBS	4.1	299.0	80.0 98.9	− 55 − 55	− 25 − 25
L-PFHxS	6.1	399.0	80.0 98.9	− 74 − 74	− 30 − 30
L-PFOS	7.4	499.0	98.9 80.0	− 108 − 108	− 22 − 22
L-PFDS	8.3	599.0	80.0 98.9	− 118 − 118	− 25 − 25
Replacement PFAS					
HFPO-DA	5.3	329.0	169.0 285.0	− 18 − 8	− 11 − 11
ADONA	6.0	377.0	85.0 251.0	− 37 − 29	− 15 − 15
9Cl-PF3ONS	7.7	531.0	351.0 83.0	− 25 − 80	− 15 − 20
11Cl-PF3OUdS	8.6	631.0	451.0 83.0	− 25 − 80	− 15 − 20
Perfluoroalkylethers					
PF4OPeA	2.5	229.0	85.0 197.0	− 30 − 19	− 15 − 13
PF5OHxA	4.0	279.0	85.0 235.0	− 30 − 11	− 15 − 16
PFEESA	4.7	315.0	135.0 69.0	− 22 − 60	− 11 − 17
3,6-OPFHpA	4.8	295.0	201.0 85.0	− 16 − 29	− 7 − 15
Internal standards					
MPFBA	1.9	217.0	172.0	− 15	− 14
MPFHxA	4.9	315.0	270.0	− 12	− 5
MPFOA	6.7	417.0	372.0	− 14	− 7
MPFNA	7.3	468.0	423.0	− 14	− 7
MPFDA	7.8	515.0	470.0	− 16	− 5
MPFUdA	8.2	565.0	520.0	− 18	− 7
MPFDoA	8.7	615.0	570.0	− 18	− 7
MPFHxS	6.1	403.0	103.0	− 74	− 30
MPFOS	7.4	503.0	80.0	− 108	− 22

### Method validation

The method validation was performed during three non-consecutive days, with three calibration curves for each working session, according to a protocol previously published [30].

Parameters obtained by the nine replicates of the calibration curve were as follows: calibration, limit of detection (LOD) and limit of quantification (LOQ), intra- and inter-day accuracy and precision. Additional experiments were executed for matrix effect, recovery, and stability assessment. For specificity evaluation, potentially jeopardized by the presence of interfering signals from the blood matrix, four samples, three obtained by non-exposed volunteers and one resulting from the pooled whole blood used for validation, were analyzed in two conditions, either adding the internal standards mixture or without it.

#### Calibration

The data used to build the calibration curves were obtained by analyzing extracts of *Capitainer*®*B* cards loaded with whole blood spiked at the following concentration levels: 2–5-10–20-50–100 ng/mL. The cards were then treated and analyzed as described in “Sample preparation” and “Instrumentation.”

The calibration model was generated for each analyte in the 2–100 ng/mL range by calculating the peak-area ratios between the target analyte and the associated internal standard, then plotting them on the y-axis against the six concentrations ratios between each concentration level and internal standard concentration on the x-axis.

Homoscedastic vs. heteroscedastic data distribution was evaluated by examining the variance of nine data points at six concentration levels. If the variance increases with the concentration, heteroscedasticity was proven (*p* < 0.05). In this case, a weighted model, 1/x or 1/x^2^, was adopted depending on the linear or quadratic increase of the variance with concentration, respectively [31]. The order of the calibration model, linear or quadratic, was generated based on the results of Mandel and lack-of-fits tests.

#### LOD and LOQ

The limit of detection (LOD) was calculated using the Hubaux-Vos equation on the linear portion of the calibration curve (i.e., the four lowest calibration points were considered), adjusted for data heteroscedasticity using Currie’s weighting correction [32]. The resulting LOD values were subsequently tested experimentally, by spiking the blank matrix at three concentration values around the calculated LOD values and verifying that the signal-to-noise ratio (S/N) was higher than 3. The limit of quantification (LOQ) was assigned to the lowest calibration level, for which acceptable accuracy and precision criteria (within the ± 20% range and below 20% respectively) were fulfilled.

In view of the method application to PFAS exposure monitoring for a large cohort of individuals, further experimental evaluations were conducted to stress the achievable detection limits, by considering both a double extraction option on a single card and the combination of the two available spots collected by the *Capitainer*®*B* card from two blood samples loading.

To this purpose, the following experimental settings were prepared in three replicates at 2 ng/mL (first point of the calibration curve):

i. Double extraction procedure applied to one spot containing 10 μL of blood;
ii. 20 μL of blood derived from two spots processed by the same extraction protocol developed for one spot;
iii. Double extraction carried out on two spots at once.

Afterwards, the 9 solutions obtained thereby were injected into the UHPLC-MS/MS system, and LOD values were calculated by extrapolating a S/N = 3 referred to the lowest MS/MS transition for each analyte. The average value was considered for each set of three replicates. The choice of the lowest transition was not possible for PFBA and PFPeA whose detection is based on one transition only.

#### Accuracy and precision

Intra- and inter-day accuracy were expressed as bias% for each concentration level. The intra-day accuracy was calculated considering for each analytical session the calibration model built from two repeated experiments and performing a back-calculation to obtain the six data points of the third curve. This process was repeated cyclically for three working days [30].

The inter-day accuracy was calculated similarly, except that the calibration model was cyclically built from the six data points at each level obtained in two working sessions, and the data points of the third day were back-calculated from them. Again, the results are averaged to provide an inter-day bias% for the six levels.

The intra-day precision was independently calculated for the 3 days of analysis; then, the coefficient of variation (CV%) was expressed as the average of precision of three analytical sessions for each level. The inter-day precision followed a similar procedure, using all nine replications collected during all three days [30]. The analytical method was assumed as validated if the mean bias% value was within the ± 20% range for all calibration levels, and the mean CV% value was below 20%.

#### Matrix effect, extraction recovery, and process efficiency

The matrix effect (ME%) was assessed at three concentration levels: low (2 ng/mL), medium (15 ng/mL), high (75 ng/mL). For each level, three replicates of blank dried blood (set 1) and three replicates of spot without blood (set 2) were subjected to the extraction process. At the end of the procedure, target analytes and internal standards were added, followed by regular analysis. The ME% was calculated from the ratio between the mean of peak-area ratios (*A*_target analyte_/*A*_internal standard_) of set 1 and of set 2 for each concentration. For each target analyte, the mean ionization suppression/enhancement was computed by averaging the ME% value over all concentration levels. ME% values were considered acceptable within 85–115% range.

In case of significant ion suppression/enhancement, the standard addition method was tested as an alternative quantitation method, as is often practiced when the sample complexity results in significant matrix effects.

The extraction recovery (ER%) was evaluated at the same concentration levels (low, medium, high). Three replicates of fortified dried blood underwent the extraction protocol, at the end of which the internal standards were added (set 3). The extraction recovery was calculated by the ratio between the mean of peak-area ratios (*A*_target analyte_/*A*_internal standard_) of set 3 (addition before the extraction step) and set 1 (addition after the extraction step) for each concentration. The ER% values, averaged over the 3 concentration levels, were considered acceptable when resulted in the range 85–115%. Lastly, the overall process efficiency (PE%) was calculated by multiplying the matrix effect by the extraction recovery (ME% × ER%) [33].

#### Stability

The stability of the target analytes was investigated at low (2 ng/mL) and high (75 ng/mL) concentrations under three different storage temperatures: − 20 °C, 4 °C, and 25 °C (room temperature).

At day 0, the blood fortified by the analytical standard of target compounds was deposited onto the microsampling devices, which were subsequently stored at the temperatures mentioned above. Three storage periods were considered, 1, 14, and 28 days, and three replicates were prepared for each concentration, temperature, and time conditions (54 samples). The ratio between the peak intensity of each analyte and the internal standard was calculated for every considered condition and the variability was assessed by the statistical analysis of variance (ANOVA), using a significance *p*-value of 0.05.

The choice of specific storage conditions reflects the realistic scenarios to which real samples could be exposed, in terms of collection, transport, and (delayed) processing [34].

### Optimization of the extraction process

An explorative full-factorial experimental design (DoE) was initially built to optimize the extraction process. In particular, the volume of the extraction solvent (methanol) and the sonication time were investigated at the following setting levels:

1. 250, 500, 750 μL of methanol
2. 10, 20, 30 min of sonication

resulting in nine experiments (2 variables, 3 levels: 3^2^ experiments), executed in random order at the fixed concentration of 5 ng/mL, as outlined in Table 2.

**Table 2:** Volume of extraction solvent and sonication time are reported for each experimental point

Experiment	MeOH volume (μL)	Sonication time (min)
9	750	30
7	250	30
5	500	20
3	750	10
8	500	30
4	250	20
1	250	10
2	500	10
6	750	20

From the peak area ratio recorded for the 25 target analytes, the statistical analysis considered in turn: only the amount of solvent, only the sonication time, and the two combined variables. The data were initially explored by principal component analysis (PCA) to evaluate the overall variability of the collected results for the 25 molecules. Then, a multiple linear regression (MLR) model was built to investigate the relevance of the investigated parameters in the extraction process.

Two final sets of experiments were conducted to investigate the conditions under which the calibrators are prepared. In particular, two different drying times were compared and the possible addition of an equilibration step for the fortified blood was evaluated at six concentration levels.

### Whole blood versus plasma sampling

In order to estimate the PFAS distribution between the cellular component of blood and its plasma fraction and investigate the potential bias that may arise therefrom, some comparative experiments were conducted on dried spots loaded with either spiked blood or spiked plasma.

Plasma was obtained from pooled whole blood by centrifugation at 4000 rpm for 15 min. After the separation of the two phases, 100 μL of plasma was dispensed into 2 mL PP Eppendorf vials, and the appropriate volume of spiking solution was added to achieve the same six concentration levels used for the calibration curve. The fortified plasma was also spotted in the inlet of the *Capitainer*®*B* cards, which was left to dry at room temperature for 3 h. Then, the same extraction procedure used for DBS cards was applied.

Parallel fresh calibration curves were prepared from both spiked blood spots (DBS) and spiked plasma spots (DPS), as described in “Calibration.” Moreover, parallel DBS and DPS samples were prepared at 15 ng/mL and 30 ng/mL for all target analytes. Before loading the spiked matrices onto the microsampling device, both blood and plasma were subjected to an equilibration delay of 24 h at 4 °C in order to mimic a real specimen acquired from a human subject. PFASs determination was performed in the following way:

1. The concentration in the DBS samples was quantified using the corresponding DBS calibration curve, and afterward the DPS calibration curve;
2. The DPS samples were treated similarly; quantification was assessed by the DPS curve and then matched with the results obtained using the DBS calibration curve.

## Results

Traces of the analytes PFBA, PFOA, PFNA, and L-PFBS were detected (> LOD) in both the pooled blood collected for the method development and validation from *non-exposed* volunteers and also the DBS cards loaded with capillary blood from the same volunteers, but the detected levels were considered negligible because they were below the LOQ. In contrast, the extraction solvent, injection vial cap, and reconstitution solvent did not show any signals related to the PFAS presence.

**Fig. 1:**
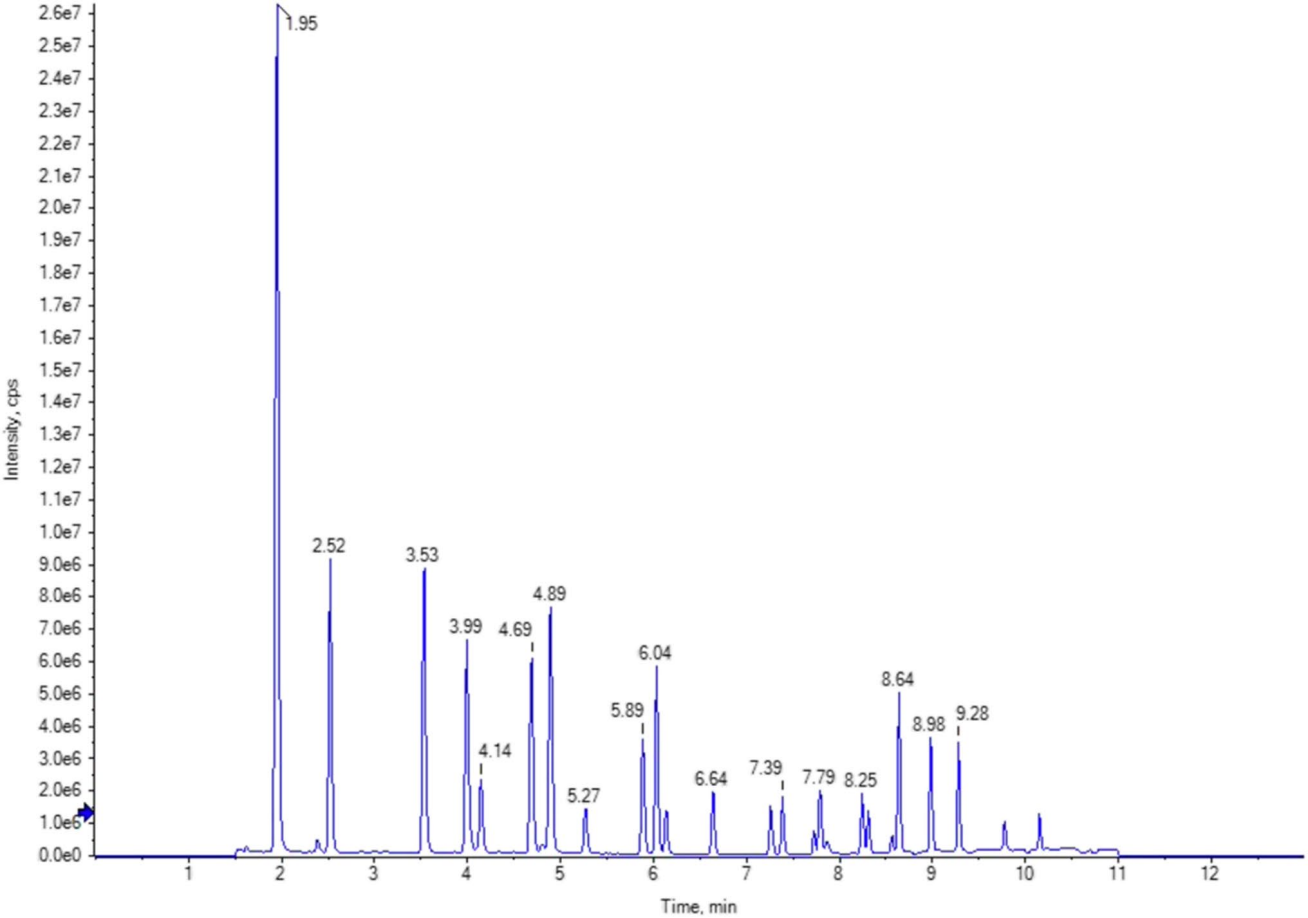
Chromatographic profile of 25 PFAS target analytes

The total ion chromatogram (TIC) of a 20 ng/mL calibrator is reported in Fig. 1, where 25 chromatographic peaks are visible, corresponding to the target molecules. The decreasing trend showed by signal intensities at increasing retention time (and the number of carbon atoms in the molecular structure) roughly corresponds to the decrease of the analyte concentration, expressed in pmol/mL, with their increasing molecular weight. The identification of each molecule based on its retention time was unambiguous since no coelution of analytes occurred. The addition of internal standards did not produce any difference in the retention time of the target analytes.

### Optimization of the extraction process

In quantitative methods based on dried blood spots, the pre-analytical conditions, and in particular the extraction procedures, are of crucial importance, since potential contaminants extracted from the blood matrix or filter paper materials might affect the ionization efficiency and signal intensity [35].

The DBS sample treatment is usually performed by extraction assisted by ultrasonication and/or centrifugation. The pivotal parameters for an optimized extraction procedure are the choice of the extraction solvent and possible modifiers, the required volume of solvent, the kind of assistance needed, temperature, and time factors [36]. Previous studies recognized methanol as a high-performance solvent, guaranteeing the extraction of a broad panel of PFAS [37] that the present study confirms. Ultrasonication at room temperature and centrifugation were implemented to assist the extraction process. In addition, two parallel procedures were compared: the first involved the direct injection of the extraction solution, while the second included an evaporation step and reconstitution with either methanol or the mobile phase solution. The latter approach (evaporation and reconstitution with the extraction solvent) provided the best signal intensity for each target compound and a high signal-to-noise ratio, even at low analytes’ concentration.

**Fig. 2:**
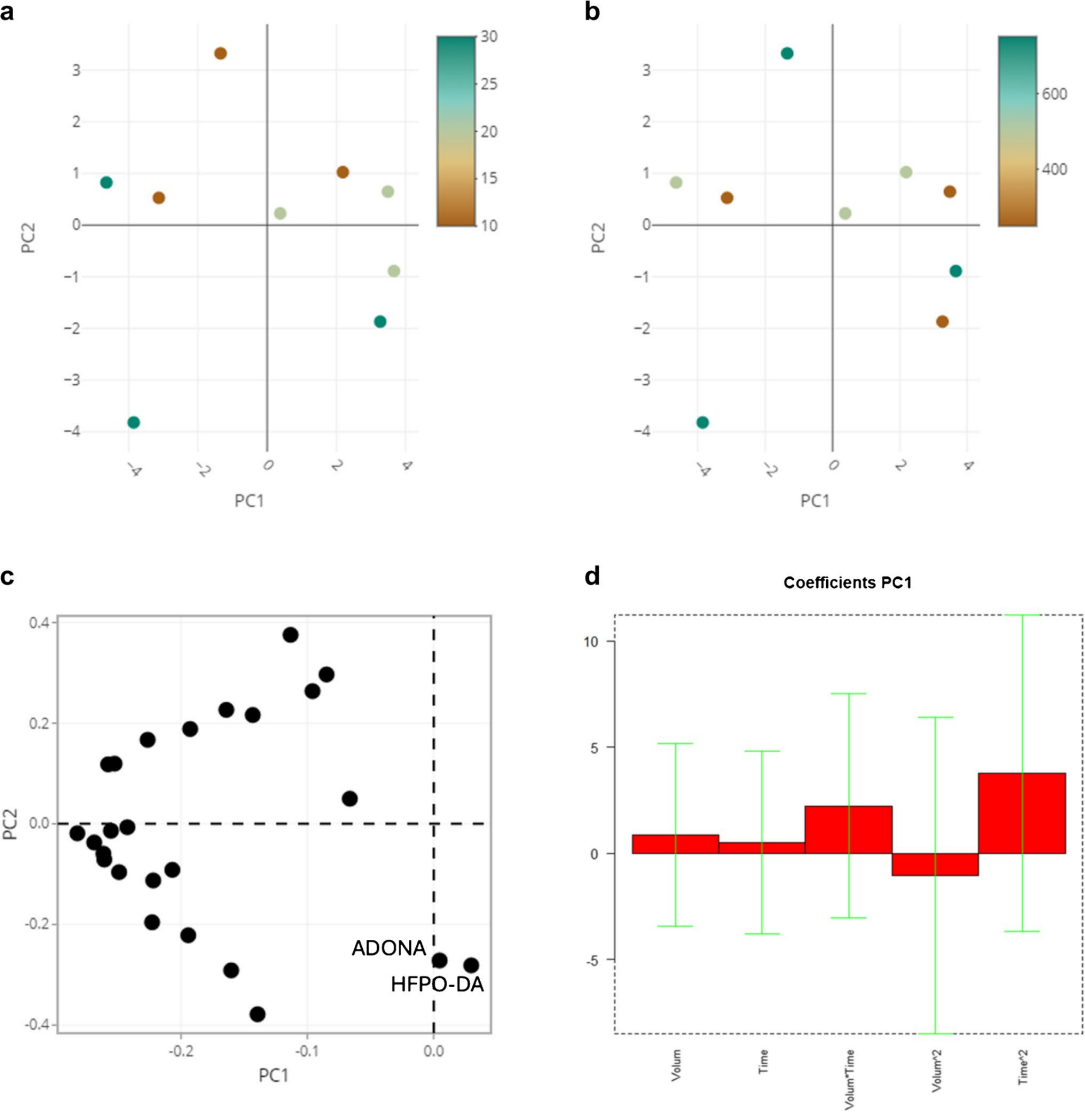
Scores plots of the PCA model colored using **a** the sonication time and **b** the methanol volume; **c** loading plot of the PCA model; **d** coefficients of the MLR regression model

The DoE experiments provided a large dataset (9 experiments × 25 analytes), which was initially explored by PCA. The score plot for a representative panel of 9 analytes (PFHxA, PFOA, PFNA, L-PFHxS, L-PFOS, HFPO-DA, ADONA, PF4OPeA, PF5OHxA) is reported in Fig. 2a–b, with different coloring for sonication time (Fig. 2a) and methanol volume (Fig. 2b). Due to statistical match observed in the preliminary results among analytes with similar structure, the 9 analytes are selected as representative of the class they belong to. Higher extraction values for the 25 analytes—on average—are distributed along the negative direction of PC1, except for ADONA and HFPO-DA. In contrast, neither the methanol volume nor the sonication time turned out significant by combining the score plot with the loading plot as reported in Fig. 2c. The same conclusion was drawn from the MLR coefficients of the DoE model built using the PC1 score values as the dependent response of the regression model (Fig. 2d). Despite a common trend of most analytes to yield higher signals as both parameters are increased, the observed difference between 500 and 750 μL methanol is minimal. Thus, the designated extraction volume was 500 μL, according to the sustainability concept of using less volume of solvents [38], while the sonication time was set to 30 min since this parameter showed a modest yet clearly detectable influence on the average extraction yield.

No significant differences were recorded by either allowing the blood spots to dry overnight or just for 3 h after deposition of spiked blood on the microfluidic device. Thus, the latter condition was adopted, as also suggested by the literature.

### Calibration, accuracy, precision, LOD, and LOQ

Regression models were obtained for each molecule of the selected panel using the six concentration levels and nine replications collected in three non-consecutive days (54 analysis). The variance analysis at low, medium, and high concentrations demonstrated a heteroscedastic data distribution. A 1/x^2^ weighting factor was adopted since the variance increased with concentration according to quadratic proportion. The choice between linear or quadratic calibration models was decided by the results of Mandel and lack-of-fits tests: for twenty analytes a linear model was selected, while a quadratic model resulted for PFNA, L-PFHxS, L-PFOS, L-PFDS, and 9Cl-PF3ONS.

Table 3 shows the calculated calibration parameters (equation and model) and reports the corresponding internal standards and the LOD values calculated by the Hubaux-Vos method. For the molecules not having a corresponding ^13^C-mass labelled internal standard available, the matching was made on the basis of the molecular structure similarity and the fitness of the retention time.

**Table 3:** Calibration parameters (equation and model) selected internal standard, and limit of detections calculated by the Hubaux-Vos method for the target PFAS analytes

Analyte	Equation	Model	ISTD	LOD (ng/mL)
Perfluorocarboxylic acids				
PFBA	*y* = 0.8092*x* + 0.0579	Linear	MPFBA	0.6
PFPeA	*y* = 0.3435*x* + 0.0068	Linear	MPFBA	0.7
PFHxA	*y* = 1.103*x* + 0.122	Linear	MPFHxA	0.7
PFHpA	*y* = 0.7907*x* + 0.0382	Linear	MPFHxA	0.6
PFOA	*y* = 1.0968*x* + 0.1978	Linear	MPFOA	1.0
PFNA	*y* = − 0.0156*x*^2^ + 0.953*x* + 0.057	Quadratic	MPFNA	0.8
PFDA	*y* = 0.9312*x* + 0.0197	Linear	MPFDA	0.6
PFUdA	*y* = 0.9854*x* + 0.039	Linear	MPFUdA	0.6
PFDoA	*y* = 0.89*x* + 0.0311	Linear	MPFDoA	0.5
PFTrDA	*y* = 1.1157*x* + 0.008	Linear	MPFDoA	0.5
PFTeDA	*y* = 0.8752*x* + 0.013	Linear	MPFDoA	0.7
PFHxDA	*y* = 0.6599*x* − 0.0098	Linear	MPFDoA	0.6
PFODA	*y* = 0.7385*x* − 0.0222	Linear	MPFDoA	0.4
Perfluorosulfonic acids				
L-PFBS	*y* = 9.9377*x* + 0.0499	Linear	MPFHxS	0.5
L-PFHxS	*y* = − 0.0538*x*^2^ + 5.5136*x* + 0.0339	Quadratic	MPFHxS	0.4
L-PFOS	*y* = − 0.0165*x*^2^ + 1.0528 + 0,045	Quadratic	MPFOS	0.4
L-PFDS	*y* = − 0.018*x*^2^ + 1.0766*x* + 0.018	Quadratic	MPFOS	0.8
Replacement PFAS				
HFPO-DA	*y* = 0.1406*x* + 0.0051	Linear	MPFHxA	0.7
ADONA	*y* = 3.236*x* − 0.1108	Linear	MPFOA	0.7
9Cl-PF3ONS	*y* = 0.0144*x*^2^ + 0.7061*x* + 0.0235	Quadratic	MPFOS	0.6
11Cl-PF3OUdS	*y* = 0.4769*x* + 0.0143	Linear	MPFOS	0.7
Perfluoroalkylethers				
PF4OPeA	*y* = 0.4607*x* + 0.0111	Linear	MPFBA	0.5
PF5OHxA	*y* = 1.6888*x* + 0.0302	Linear	MPFHxA	0.7
PFEESA	*y* = 36.76*x* + 0.4631	Linear	MPFHxA	0.7
3,6-OPFHpA	*y* = 0.0356*x* + 0.0001	Linear	MPFHxS	1.0

The LOD values calculated with the weighted Hubaux-Vos method ranged from 0.4 ng/mL (PFODA, L-PFHxS, L-PFOS) up to 1.0 ng/mL (PFOA, 3,6-OPFHpA). Previous studies [37, 39, 40] concerning the determination of PFAS in DBS reported lower limits of detection by an order of magnitude than those reported in the present study (Table 3), which, on the other hand, uses a smaller volume of blood (10 μL) and does not benefit from solid-phase extraction (SPE), thus reducing wastes and speeding up the sample treatment. Therefore, the LOD values achieved by the present method perfectly fit the proposed purpose of monitoring the PFAS levels in exposed and at-risk population, namely at levels above 2 ng/mL. For all the target analytes, the LOQ values correspond to the lowest concentration of the calibration curve (2 ng/mL), for which the predetermined accuracy and precision requisites are fully satisfied, as discussed below.

Full accuracy and precision data are accessible in the supplementary material (Table S1) for all target analytes at each concentration level (6 levels). Overall, the intra- and inter-day bias% values proved that the method is accurate: all bias% values fall in the range from − 20 to + 20%, demonstrating the reliability of the developed method in the determination of PFAS levels in a wide range of concentrations, including the lowest (LOQ). The mean bias% values, averaged for the 6 concentrations, were below the absolute value of 10% for all analytes with the only exceptions of PFOA (− 13% as bias intra-day) and PFUdA (+ 12% as bias inter-day). No significant differences were detected for analytes differing for chain length, type of polar head, and presence or absence of ether group, with a random distribution of bias% values observed in the ± 10% range. Inter-day accuracy was on average only slightly lower than intra-day accuracy, showing an adequate degree of trueness in the quantification performed during different working days and along rather extended periods of time.

Also, the intra- and inter-day precision, expressed as CV%, provides evidence of good reproducibility both during a single working day and in separated sample preparation sessions. All single CV% values recorded for each analyte and level ranged from 1 to 19%, while the concentration-averaged values ranged from 3% (L-PFOS intra-day precision) to 15% (HFPO-DA inter-day precision). Unlike the accuracy data, inter-day averaged CV% values proved moderately higher than intra-day CV%, with few non-significant exceptions (PFUdA, 5% inter-day and 8% intra-day CV%; 3,6-OPFHpA, 12% inter-day and 13% intra-day). Notably, acceptable bias% and CV% results were obtained at the lowest calibration level, confirming 2 ng/mL as LOQ for all 25 target analytes.

### Limits of detection re-evaluation

The *Capitainer®B* card presents two microfluidic channels, which allow to collect two calibrated blood samples of 10 μL each on two paper discs. This makes it possible to treat only one quantitative sample or both spots simultaneously, either with single or double extraction, as described in the experimental section. The data produced by these extra experiments are reported in Table 4.

**Table 4:** LOD values obtained from three experimental settings, as extrapolated (S/N = 3) from the average S/N value from three DBS cards replicates spiked at 2 ng/mL analytes concentration

Analyte	LOD (ng/mL) 10 μL (1 spot) Double extraction	LOD (ng/mL) 20 μL (2 spots) Single extraction	LOD (ng/mL) 20 μL (2 spots) Double extraction
PFHxA	0.24	0.14	0.19
PFHpA	0.11	0.26	0.07
PFOA	0.03	0.02	0.08
PFNA	0.01	0.01	0.01
PFDA	0.23	0.16	0.12
PFUdA	0.13	0.10	0.13
PFDoA	0.30	0.28	0.40
PFTrDA	0.31	0.22	0.24
PFTeDA	0.30	0.17	0.30
PFHxDA	0.30	0.33	0.15
PFODA	0.30	0.37	0.27
L-PFBS	0.20	0.16	0.13
L-PFHxS	0.18	0.12	0.20
L-PFOS	0.32	0.18	0.38
L-PFDS	0.30	0.60	0.30
HFPO-DA	0.40	0.18	0.28
ADONA	0.04	0.02	0.07
9Cl-PF3ONS	0.40	0.40	0.30
11Cl-PF3OUdS	0.40	0.40	0.30
PF4OPeA	0.30	0.40	0.38
PF5OHxA	0.34	0.40	0.40
PFEESA	0.19	0.08	0.09
3,6-OPFHpA	0.50	0.31	0.38

The limits of detection achievable by these expanded procedures are clearly lower than the LOD values calculated by the Hubaux-Vos algorithm during the validation protocol; nevertheless, the large majority of the new LODs are within the same order of magnitude as the preceding ones, with the exception of PFOA, PFNA, and ADONA in all new settings, and PFHpA, PFUdA, and PFEESA under specific conditions. On average, the most exhaustive conditions (double disc, double extraction) produce a slight improvement (i.e., decrease) in LOD values, not substantially different from the standard conditions (one disc, single extraction). According to the epidemiological/monitoring aim of this study to evaluate PFAS levels that could induce toxic effects in exposed subjects, it can be concluded that only one spot from the *Capitainer*®*B* card is required and the double extraction process is particularly unnecessary. From the perspective of application, one card will be used to collect two samples from the same subject: one spot will be processed by the validated method, while the second one could be stored in case a confirmation analysis is requested.

### Matrix effect, extraction recovery, and process efficiency

The matrix effect experiments showed that the presence of matrix components produces a generalized ion enhancement (IE%) for almost all analytes, as reported in Table 5, where the average values deriving from three concentrations and the corresponding relative standard deviations are indicated. In particular, the signal enhancement proved substantial (> + 50%) for thirteen analytes and moderate (+ 25% < IE% <  + 50%) for other eleven analytes, with the only exception of HFPO-DA (− 16%). The highest enhancement was recorded for PFODA (+ 93%).

**Table 5:** Matrix effect (%), ion enhancement/suppression (%), recovery (%), and process efficiency (%) calculated as average value of three concentration levels for each target molecule. Relative standard deviations (RSD) are reported in brackets for ME% and ER%

Analyte	ME%	Ion enhancement or suppression%	ER%	PE%
PFBA	131% (13%)	+ 31%	98% (18%)	129%
PFPeA	180% (19%)	+ 80%	91% (18%)	163%
PFHxA	128% (12%)	+ 28%	93% (22%)	119%
PFHpA	169% (22%)	+ 69%	109% (20%)	185%
PFOA	156% (12%)	+ 56%	96% (2%)	149%
PFNA	152% (18%)	+ 52%	82% (21%)	125%
PFDA	178% (21%)	+ 78%	98% (23%)	175%
PFUdA	162% (8%)	+ 62%	102% (18%)	165%
PFDoA	149% (17%)	+ 49%	96% (22%)	144%
PFTrDA	130% (12%)	+ 30%	83% (5%)	108%
PFTeDA	171% (19%)	+ 71%	76% (7%)	131%
PFHxDA	145% (19%)	+ 45%	77% (23%)	112%
PFODA	193% (16%)	+ 93%	89% (15%)	171%
L-PFBS	178% (18%)	+ 78%	84% (8%)	178%
L-PFHxS	131% (23%)	+ 31%	90% (7%)	131%
L-PFOS	127% (21%)	+ 27%	92% (16%)	116%
L-PFDS	148% (13%)	+ 48%	84% (2%)	124%
HFPO-DA	84% (10%)	− 16%	103% (16%)	84%
ADONA	142% (8%)	+ 42%	90% (19%)	128%
9Cl-PF3ONS	169% (9%)	+ 69%	99% (20%)	167%
11Cl-PF3OUdS	186% (18%)	+ 86%	101% (8%)	187%
PF4OPeA	166% (10%)	+ 66%	96% (13%)	159%
PF5OHxA	167% (5%)	+ 67%	94% (18%)	157%
PFEESA	138% (19%)	+ 38%	98% (11%)	134%
3,6-OPFHpA	143% (22%)	+ 43%	92% (17%)	132%

Apparently, these results cannot be attributed to the molecular structure of the analytes, since no trend could be associated with the number of carbon atoms in the alkyl chain or the terminal polar group. As reported by Lin et al. [40], the notable matrix effect observed in the present study could be attributed to the blood drying step, since it was not detected in previous works focused on PFAS determination on plasma, serum, or whole blood [41]. However, the intensity of matrix effects does not depend only on the matrix itself but also on the experimental conditions under which electrospray ionization is operated. Since these effects influence the analyte quantification, it is recommended to control them carefully in any method validation protocol.

The recovery results (Table 5), arising from the comparison between the concentration measured after spiking the analytes respectively before and after the extraction step, ranged from 77% (PFHxDA) to 109% (PFHpA). Six analytes (PFNA, PFTrDA, PFTeDA, PFHxDA, L-PFBS, L-PFDS) showed values slightly below 85%, which corresponds to the minimum value of acceptability. However, taking into account the large panel of 25 target molecules, they can be considered satisfactory. These data represent a fundamental confirmation of the efficiency of the sample preparation method in recovering a broad range of PFAS compounds from a complex substrate (i.e., dried blood on a solid support). In this support, cellulose is one of the main components: due to the polar PFAS terminal groups, a polar-polar interaction between the end-tail functional group and the cellulosic substrate is possible [42]. Therefore, the high recovery results demonstrated that methanol and ultrasonic shaking represent adequate conditions to release these interactions and to ensure good extraction efficiency.

The relative standard deviation (RSD) calculated for the matrix effect and the extraction recovery (Table 5) are mostly below 20%, which is considered the acceptability limit for bioanalytical methods. However, ten values are slightly higher than this limit, with the maximum reported RSD corresponding to 23%. Considering the broad range of target molecules and the wide concentration range tested, the RSD values can still be considered suitable.

The overall process efficiency, estimated by combining matrix effects with recovery (Table 5), resulted in PE% values within the 112–185% range, of which the major contribution was provided by the ion enhancement effect.

### Stability

**Fig. 3:**
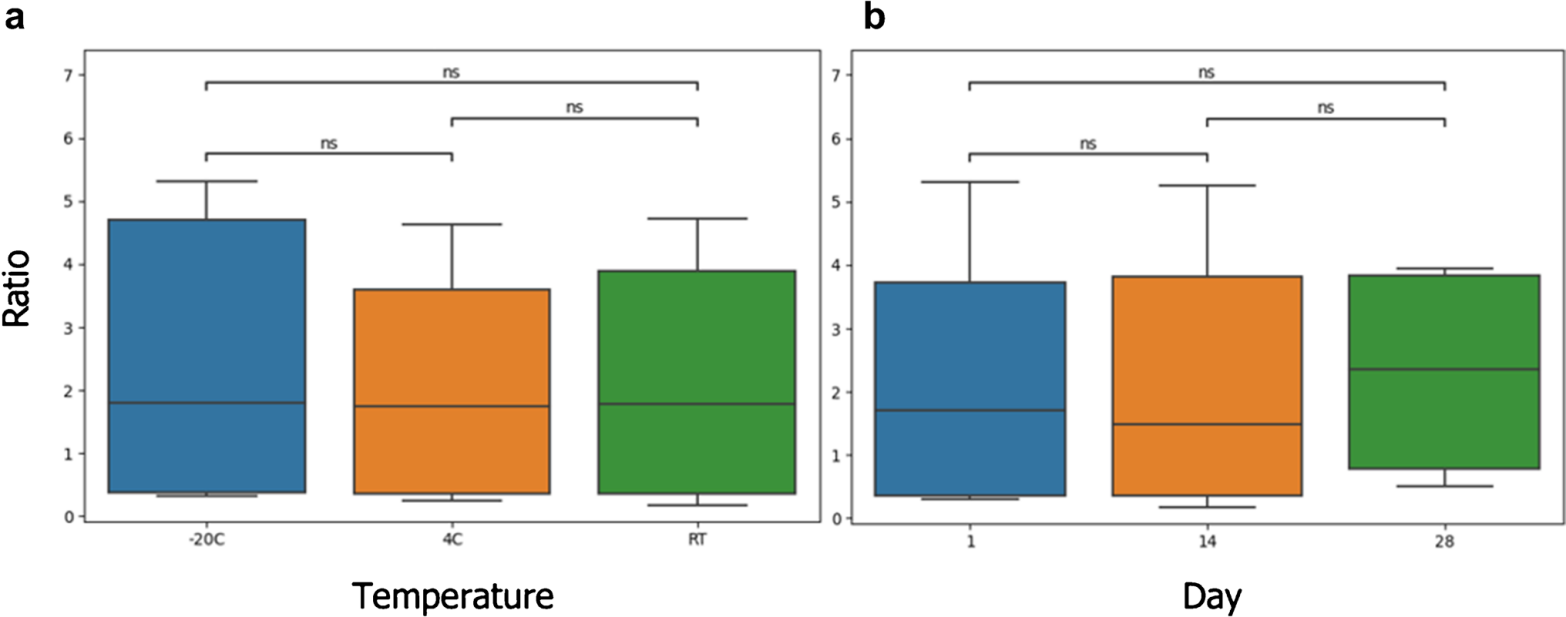
Boxplots as graphical representation of ANOVA performed by varying temperature (**a**) and time (**b**) parameters for PFOA

The stability of 25 PFAS within the DBS matrix was evaluated by varying the storage time (1, 14, 28 days) and temperature (− 20 °C, 4 °C, 25 °C) at low and high concentrations. The results are reported in Fig. 3. In general, all the analytical results obtained from the different experiments and samples proved comparable. Taking into account the variance observed in repeated experiments, no significant difference (*p* < 0.05) was evidenced in the mean values obtained from the nine conditions tested.

It is concluded that all analytes are stable in the DBS samples for at least 4 weeks regardless of the storage temperature, as is expected for substances whose stability and persistence in the environment and biological organisms is the source of concern. From the perspective of real sample analysis, these experimental results demonstrated that DBS collected on *Capitainer*®*B* cards can be stored at room temperature without need of rapid and timely processing.

### Dried blood spot–dried plasma spot comparison

This study is aimed at PFAS determination in DBS, generally assimilated to whole blood. Previous studies conducted on plasma and serum demonstrated that PFAS tend to accumulate in body compartments with protein-rich contents, in particular, they strongly bind to serum proteins [23]. For this reason, it was assumed that these compounds are distributed mainly into the liquid component of blood and only to a limited extent into the cellular components. Thereby, considering that plasma constitutes approximately 55% of whole blood, the measured PFAS concentration should be approximately twice in plasma and serum than in whole blood [15]. However, different PFAS show distinct physicochemical properties, from which different binding affinities to proteins are expected [20]. In the toxicological evaluations of exposed populations, awareness about their distribution among different blood components might be of importance.

To clarify this aspect, experiments were conducted in parallel, loading the sampling cards with either spiked whole blood (WB-S) or spiked plasma (P-S) and quantifying the PFAS with two calibration curves built from either blank whole blood (DBS-cal) or blank plasma (DPS-cal). The quantitative data obtained from DBS and DPS spiked with 15 ng/mL and 30 ng/mL PFAS are reported in Table S2 of the supplementary material.

Inaccurate results are obtained only for perfluorocarboxylic acids with long perfluoroalkyl backbone (number of carbons > 12) on DPS samples at both 15 ng/mL and 30 ng/mL concentrations when the quantification is performed by DBS calibration. Specifically, underestimation in these samples for four target analytes may reach 50%. All the other quantitative results appear to be accurate, no matter if the calibration curve was built on a matrix different from the sample. In particular, all the quantitative results showed reasonable accuracy when the calibration curve was built from spiked plasma samples (DPS-cal). For 21 analytes, quantification of fortified whole blood and plasma samples can be achieved by DBS and DPS calibration curves without significant differences, while for long-chain perfluorocarboxylic acids the use of a calibration curve in the corresponding matrix is recommended. These conclusions should be reconsidered once real DBS specimens are available.

### Blue applicability grade index

Recently, to gauge the applicability and viability of methods, the blue applicability grade index (BAGI) was introduced as a specific metric tool that provides a quantitative approach through the scores assigned to ten attributes of equal importance [43]. Specifically, the blueness refers to how fit-for-purpose a technique is in real-world applications. To date, several green metric tools have been implemented to assess the green performance of an analytical method and its impact on the environment, but they do not consider practicability, a dominant requirement encountered by routine analysis laboratories [44].

The performance characterization of key parameters is reported below for the present method:

i. Quantitative analysis;
ii. Multi-element analysis for 25 compounds belonging to four classes;
iii. UHPLC-MS/MS instrumentation;
iv. The simultaneous preparation of 13–95 samples was estimated as viable;
v. Multi-step preparation is required;
vi. In 1 h, any sample can be analyzed because of the time required for sonication, centrifugation, evaporation, and reconstitution steps;
vii. Reagents used are commercially available;
viii. Preconcentration is accomplished through extraction, evaporation, and reconstitution;
ix. The UHPLC-MS/MS system provides automatic injection and semi-automatic detection;
x. The amount of biological sample is < 100 μL (10 μL of capillary blood).

**Fig. 4:**
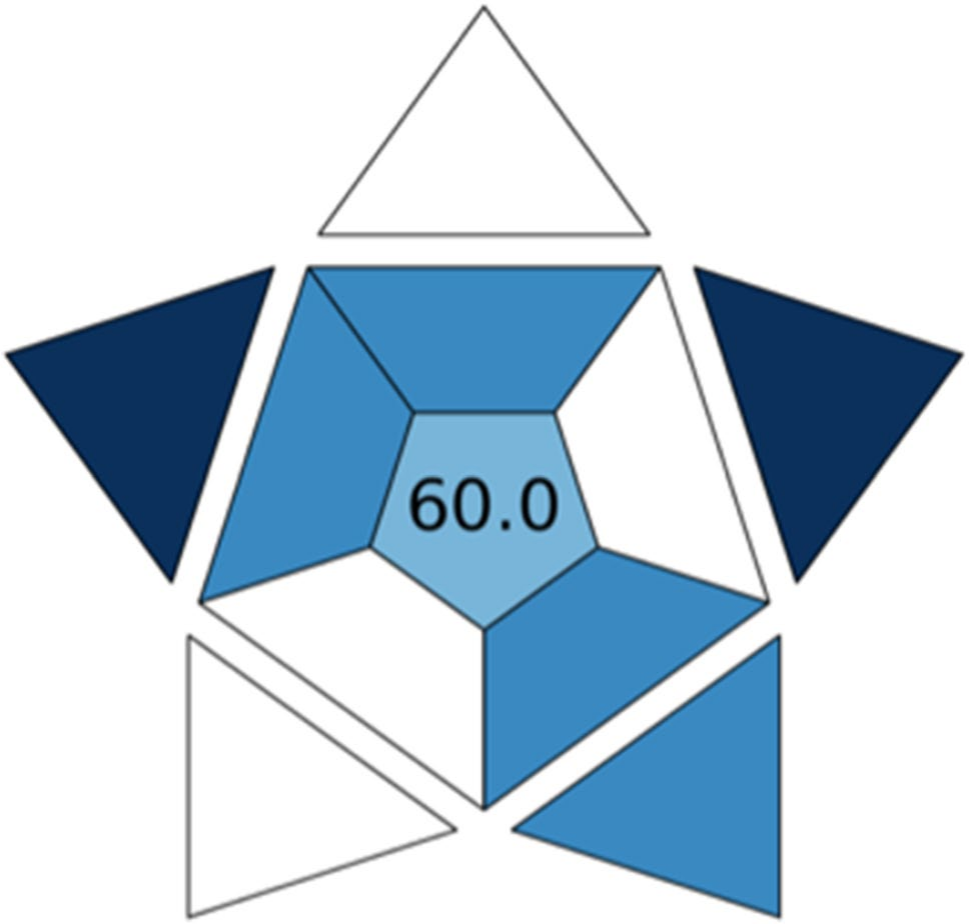
BAGI pictogram: the different shades of blue depend on assigned scores

A final score ranging from 25 to 100 points was calculated. Figure 4 shows the obtained BAGI pictogram with the correlated final score.

The four attributes to which the white color was assigned (low score) concern the complexity of the extraction procedure and instrumentation. The four attributes displaying the light blue color (medium score) are related to the type of analysis, number of target analytes, simultaneous sample preparation, and automation degree. The two factors in dark blue (high score) indicate the needed reagents and the amount of sample.

In order to be considered “practical,” the method should attain a recommendable score of at least 60 points [43]. The proposed method obtained a score corresponding to the minimal acceptance threshold. The weak aspects emphasized in terms of applicability to real routine sample analysis are referred to the multi-step sample preparation, requiring preconcentration to achieve sufficient sensitivity, time to process samples, and relatively expensive instrumental equipment. Thereby, strategies to reduce the number of steps and the turnaround time will be explored in future improvements of the method performance.

## Discussion

The developed and validated method proved its effectiveness for human blood analysis for PFAS, which in turn opens encouraging scenarios for extensive monitoring in at-risk populations, such as exposed workers, newborns, and pregnant women. Notably, this workflow aims to contribute to epidemiological studies to assess the exposure levels to a category of “forever chemicals,” which has been gaining increasing public concerns and media attention only in the recent period. Prospectively, estimating PFAS concentrations in human blood will be helpful in understanding which kind of precautions should be taken in order to limit as much as possible the adverse health effects. Therefore, from this point of view, the concentration interval chosen for the quantification range (from 2 to 100 ng/mL) considers 2 ng/mL as the threshold above which in-depth clinical tests appear to be necessary.

In this context, DBS is an attractive and reliable sampling method to evaluate the individual *exposome.* Recently, the characterization of human *exposome* has gained growing interest to prevent conceivable health outcomes as a result of environmental exposures to persistent and bioaccumulating organic compounds [45]. Nowadays, emphasis is directed toward sampling technologies involving minimally invasive biological sample collection and low-volume storage and handling [38]. In this perspective, the dried blood spot microsampling technique may constitute a reasonable alternative to whole blood collected by traditional venous withdrawal. Indeed, DBS sampling is feasible in workplaces without the need for trained health care personnel and even at home by self-sampling.

The selected device *Capitainer*®*B* card provides a quantitative sampling that avoids the hematocrit bias [46], i.e., the quantification is not dependent on each subject’s hematocrit value. Furthermore, once the cards are sealed in an appropriate container, namely a drying pouch designated to protect and support the sample drying, the sample shipping can take place at room temperature without any analyte degradation, as the stability experiments performed in this study demonstrated.

As in common practice, venous blood collected into containers with anticoagulants was employed for the method development and validation. In order to prove its feasibility to biological samples collected by finger pricking, several *non-*exposed subjects, whose blood was used for the validation, provided DBS samples by self-collected capillary blood. These specimens were analyzed, resulting in concentrations under the LOQ for the four analytes previously identified in venous blood. Moreover, a study based on VAMS devices [39] demonstrated that the PFAS quantifications between dried venous whole blood and dried capillary whole blood are absolutely comparable.

The choice of the analytes included in this study was intended to cover a broad range of PFAS potentially subjected to human body incorporation. The panel is composed of short- and long-chain molecules belonging to carboxylic and sulfonic categories, and ethers. Most of them are designated as *legacy* PFAS, since strict regulations have been adopted in many countries around the world to phase out these molecules from industrial production [47]. On the contrary, *replacement* PFAS are compounds synthetized to replace some *legacy* PFAS. This group includes long-chain precursors of *legacy* PFAS (e.g., HFPO-DA), which are believed to be a safer alternative in terms of toxicity by producers [48].

The experimental data arising from the method validation highlighted the reproducibility and the accuracy in the quantification within the 2–100 ng/mL range of 25 selected PFAS in human blood starting from only 10 μL of sample. To our knowledge, only one other study developed a method to analyze PFAS in the same blood volume, employing a different device (VAMS) and considering a narrower panel of analytes (12 compounds) [49].

The LOD values reached in the present method are in agreement with the warning levels reported in the literature. Actually, literature studies occasionally report blood PFAS concentrations, measured in different elected cohorts, below 1 ng/mL, but these levels are declared not to be of concern.

Regarding the practical application of the method, the BAGI tool calculated the lowest acceptable score (60 points), even though the extraction recovery proved effective. Hence, an implementation of the method is foreseen so as to make sample preparation faster and decrease the sample handling steps.

Particular attention should be paid to the matrix effect, which is often associated with matrix interferents, whereby inaccurate quantifications, irreproducible results, and altered LOD and LOQ values may result [50]. A recent thoroughly conducted study demonstrated that matrix effects can be much more severe than is commonly reported [51]. Since we verified that the matrix effects are substantial, yet quite reproducible, it is important to experimentally verify their extent and in case correct the quantitative results accordingly. An effective but laborious way to compensate for consistent matrix effects in real samples is to apply the standard addition method, while using the same calibration curve built as described in the present study. During this study, the standard addition method was successfully applied to the four analytes detected at the LOD level in the pooled blood and confirmed in the capillary blood collected on DBS cards from the same individuals: the concentrations resulting from the standard addition method proved lower than the LOQs and comparable with the those obtained from the calibration curve method. The results illustrated in chapter 3.6 and reported in Table S2 also demonstrated the accurate quantification of the samples spiked at 15 ng/mL and 30 ng/mL by using the calibration curve previously built.

Concerning the impossibility of finding blank blood totally negative for PFAS, different whole blood samples from *non-exposed* volunteers were examined. Few analytes were identified, all resulting below the LOQ level: during quantification using fortified blood, their signal intensities were normalized as background. In the end, a pool of whole blood was prepared to minimize inter-individual variability owing to the matrix itself.

Particular attention should be devoted to PFBA and PFPeA analysis because their identification is only based on one transition. Consequently, the guidelines suggest to use HRMS instrumentation for the confirmation [52, 53].

The novelty of this research is that the new method proposed for PFAS monitoring in human blood requires only 10 μL of capillary blood, collected by an easy-to-handle microfluidic technology. In fact, so far very few studies focused on the determination of PFAS on DBS, and most of those used non-quantitative devices [40, 54, 55], which require supplementary considerations (related to volume and hematocrit) to provide a reproducible and accurate result.

## Conclusions

The proposed analytical approach to monitor 25 PFAS levels in selected population provides a straightforward and effective solution adaptable in the laboratory routine, combining the qDBS microsampling with UHPLC-MS/MS instrumentation. Indeed, the validation results demonstrated how the developed workflow can be reliable, sensitive, and fit-for-purpose.

Despite some additional studies to explain some aspects emerged out are needed (i.e., matrix effect), the presented research evidenced the use of microsampling devices for environmental contaminants exposure very promising. Therefore, DBS constitute a highly valuable resource for epidemiological and toxicological investigations.

Moreover, through the innovative device based on a microfluidic channel offered by *Capitainer*®*B* AB (Solna, Sweden), this work pursues green and sustainable objectives limiting the necessary volume of solvents required and the amount of laboratory supplies [38].

Lastly, it is envisaged that the developed method will be applied to the real sample analysis of potentially exposed individuals.

## Supplementary Information

Below is the link to the electronic supplementary material.

Supplementary file1 (DOCX 36 KB)

## Declarations

### Source of biological material

Whole blood samples were collected from volunteers. Informed consent from each participant was obtained.

## References

[1] Buck RC, Korzeniowski SH, Laganis E, Adamsky F. Identification and classification of commercially relevant per- and poly-fluoroalkyl substances (PFAS). Integr Environ Assess Manag. 2021;17:1045–55. 10.1002/ieam.4450. 33991049 10.1002/ieam.4450 PMC9292543

[2] Gaines LGT, Sinclair G, Williams AJ. A proposed approach to defining per- and polyfluoroalkyl substances (PFAS) based on molecular structure and formula. Integr Environ Assess Manag. 2023;19:1333–47. 10.1002/ieam.4735. 36628931 10.1002/ieam.4735 PMC10827356

[3] P.A. Rice, J. Cooper, S.E. Koh-Fallet, S.V. Kabadi, Comparative analysis of the physicochemical, toxicokinetic, and toxicological properties of ether-PFAS, Toxicol. Appl. Pharmacol. 2021 422 (2021) 115531. 10.1016/j.taap.2021.115531. 10.1016/j.taap.2021.115531 33933458

[4] Gaines LGT. Historical and current usage of per- and polyfluoroalkyl substances (PFAS): a literature review. Am J Ind Med. 2023;66:353–78. 10.1002/ajim.23362. 35614869 10.1002/ajim.23362

[5] Taylor CM, Breadmore MC, Kilah NL. Good practices and practical considerations for research with perfluoroalkyl substances. Chemistry-Methods. 2024;4: e202300017. 10.1002/cmtd.202300017.

[6] N.M. Brennan, A.T. Evans, M.K. Fritz, S.A. Peak, H.E. von Holst, Trends in the regulation of per- and polyfluoroalkyl substances (PFAS): a scoping review, Int. J. Environ. Res. Public. Health. 2021;18. 10.3390/ijerph182010900. 10.3390/ijerph182010900 PMC8536021 34682663

[7] Fenton SE, Ducatman A, Boobis A, DeWitt JC, Lau C, Ng C, Smith JS, Roberts SM. Per- and polyfluoroalkyl substance toxicity and human health review: current state of knowledge and strategies for informing future research. Environ Toxicol Chem. 2021;40:606–30. 10.1002/etc.4890. 33017053 10.1002/etc.4890 PMC7906952

[8] R.A. Brase, E.J. Mullin, D.C. Spink, legacy and emerging per- and polyfluoroalkyl substances: analytical techniques, environmental fate, and health effects, Int. J Mol Sci. 2021;22. 10.3390/ijms22030995. 10.3390/ijms22030995 PMC7863963 33498193

[9] Lucas K, Gaines LGT, Paris-Davila T, Nylander-French LA. Occupational exposure and serum levels of per- and polyfluoroalkyl substances (PFAS): a review. Am J Ind Med. 2023;66:379–92. 10.1002/ajim.23454. 36573587 10.1002/ajim.23454

[10] Burgess JL, Fisher JM, Nematollahi A, Jung AM, Calkins MM, Graber JM, Grant CC, Beitel SC, Littau SR, Gulotta JJ, Wallentine DD, Hughes RJ, Popp C, Calafat AM, Botelho JC, Coleman AD, Schaefer-Solle N, Louzado-Feliciano P, Oduwole SO, Caban-Martinez AJ. Serum per- and polyfluoroalkyl substance concentrations in four municipal US fire departments. Am J Ind Med. 2023;66:411–23. 10.1002/ajim.23413. 35864570 10.1002/ajim.23413 PMC9859935

[11] Poothong S, Papadopoulou E, Padilla-Sánchez JA, Thomsen C, Haug LS. Multiple pathways of human exposure to poly- and perfluoroalkyl substances (PFASs): from external exposure to human blood. Environ Int. 2020;134: 105244. 10.1016/j.envint.2019.105244. 31711019 10.1016/j.envint.2019.105244

[12] Zodrow J, Vedagiri U, Sorell T, McIntosh L, Larson E, Hall L, Dourson M, Dell L, Cox D, Barfoot K, Anderson J. PFAS Experts Symposium 2: PFAS Toxicology and Risk Assessment in 2021—Contemporary issues in human and ecological risk assessment of PFAS. Remediat J. 2022;32:29–44. 10.1002/rem.21706.

[13] Tian Y, Xu C, Zhang L, Shi D, Cappelli F, Yin S. Maternal exposure to per- and polyfluoroalkyl substances: implications for intrahepatic cholestasis of pregnancy and adverse birth outcomes. Expo Health. 2024. 10.1007/s12403-023-00620-6.

[14] Zheng G, Schreder E, Dempsey JC, Uding N, Chu V, Andres G, Sathyanarayana S, Salamova A. Per- and polyfluoroalkyl substances (PFAS) in breast milk: concerning trends for current-use PFAS. Environ Sci Technol. 2021;55:7510–20. 10.1021/acs.est.0c06978. 33982557 10.1021/acs.est.0c06978

[15] Jian J-M, Chen D, Han F-J, Guo Y, Zeng L, Lu X, Wang F. A short review on human exposure to and tissue distribution of per- and polyfluoroalkyl substances (PFASs). Sci Total Environ. 2018;636:1058–69. 10.1016/j.scitotenv.2018.04.380. 29913568 10.1016/j.scitotenv.2018.04.380

[16] Jia S, Marques Dos Santos M, Li C, Snyder SA. Recent advances in mass spectrometry analytical techniques for per- and polyfluoroalkyl substances (PFAS). Anal Bioanal Chem. 2022;414:2795–807. 10.1007/s00216-022-03905-y. 35132477 10.1007/s00216-022-03905-y

[17] K. Vorkamp, A. Castaño, J.-P. Antignac, L.D. Boada, E. Cequier, A. Covaci, M. Esteban López, L.S. Haug, M. Kasper-Sonnenberg, H.M. Koch, O. Pérez Luzardo, A. Osīte, L. Rambaud, M.-T. Pinorini, G. Sabbioni, C. Thomsen, Biomarkers, matrices and analytical methods targeting human exposure to chemicals selected for a European human biomonitoring initiative, Environ. Int. 2021;146:106082. 10.1016/j.envint.2020.106082. 10.1016/j.envint.2020.106082 33227583

[18] Jacobson TA, Kler JS, Bae Y, Chen J, Ladror DT, Iyer R, Nunes DA, Montgomery ND, Pleil JD, Funk WE. A state-of-the-science review and guide for measuring environmental exposure biomarkers in dried blood spots. J Expo Sci Environ Epidemiol. 2023;33:505–23. 10.1038/s41370-022-00460-7. 35963945 10.1038/s41370-022-00460-7 PMC9375076

[19] National Academies of Sciences Engineering, Medicine, Guidance on PFAS exposure, testing, and clinical follow-up, The National Academies Press, Washington, DC, 2022. 10.17226/26156. 35939564

[20] Ehresman DJ, Froehlich JW, Olsen GW, Chang S-C, Butenhoff JL. Comparison of human whole blood, plasma, and serum matrices for the determination of perfluorooctanesulfonate (PFOS), perfluorooctanoate (PFOA), and other fluorochemicals. Environ Res. 2007;103:176–84. 10.1016/j.envres.2006.06.008. 16893538 10.1016/j.envres.2006.06.008

[21] Andres KL, Olsen GW, Krisko RM, Nunnally MC, Boeding RR, Leniek KL, Taiwo OA. An investigation of 3M Cordova, IL production worker’s per- and polyfluoroalkyl substances biomonitoring results and mortality experience. Int J Hyg Environ Health. 2024;256: 114321. 10.1016/j.ijheh.2024.114321. 38244249 10.1016/j.ijheh.2024.114321

[22] Szabo D, Marchiandi J, Green MP, Mulder RA, Clarke BO. Evaluation and validation of methodologies for the extraction of per- and polyfluoroalkyl substances (PFASs) in serum of birds and mammals. Anal Bioanal Chem. 2022;414:3017–32. 10.1007/s00216-022-03962-3. 35182167 10.1007/s00216-022-03962-3 PMC8934760

[23] Poothong S, Thomsen C, Padilla-Sanchez JA, Papadopoulou E, Haug LS. Distribution of novel and well-known poly- and perfluoroalkyl substances (PFASs) in human serum, plasma, and whole blood. Environ Sci Technol. 2017;51:13388–96. 10.1021/acs.est.7b03299. 29056041 10.1021/acs.est.7b03299

[24] D.H. Chace, N.T. Lappas, The use of dried blood spots and stains in forensic science, in: W. Li, M.S. Lee (Eds.), Dried Blood Spots, John Wiley & Sons, Inc., Hoboken, NJ, USA, 2014: pp. 140–150. 10.1002/9781118890837.ch11.

[25] Jacques ALB, Santos MK, Gorziza RP, Limberger RP. Dried matrix spots: an evolving trend in the toxicological field. Forensic Sci Med Pathol. 2022;18:86–102. 10.1007/s12024-021-00434-5. 35171452 10.1007/s12024-021-00434-5

[26] Massano M, Incardona C, Gerace E, Negri P, Alladio E, Salomone A, Vincenti M. Development and validation of a UHPLC-HRMS-QTOF method for the detection of 132 new psychoactive substances and synthetic opioids, including fentanyl, in dried blood spots. Talanta. 2022;241: 123265. 10.1016/j.talanta.2022.123265. 35121540 10.1016/j.talanta.2022.123265

[27] M.U. Thangavelu, B. Wouters, A. Kindt, I.K.M. Reiss, T. Hankemeier, Blood microsampling technologies: innovations and applications in 2022, Anal Sci Adv. 2023;ansa.202300011. 10.1002/ansa.202300011. 10.1002/ansa.202300011 PMC10989553 38716066

[28] Freeman JD, Rosman LM, Ratcliff JD, Strickland PT, Graham DR, Silbergeld EK. State of the science in dried blood spots. Clin Chem. 2018;64:656–79. 10.1373/clinchem.2017.275966. 29187355 10.1373/clinchem.2017.275966

[29] European Medicines Agency, ICH M10 on bioanalytical method validation - Scientific guideline, 2023. https://www.ema.europa.eu/en/ich-m10-bioanalytical-method-validation-scientific-guideline.

[30] Alladio E, Amante E, Bozzolino C, Seganti F, Salomone A, Vincenti M, Desharnais B. Experimental and statistical protocol for the effective validation of chromatographic analytical methods. MethodsX. 2020;7: 100919. 10.1016/j.mex.2020.100919. 32477896 10.1016/j.mex.2020.100919 PMC7248235

[31] Gu H, Liu G, Wang J, Aubry A-F, Arnold ME. Selecting the correct weighting factors for linear and quadratic calibration curves with least-squares regression algorithm in bioanalytical LC-MS/MS assays and impacts of using incorrect weighting factors on curve stability, data quality, and assay performance. Anal Chem. 2014;86:8959–66. 10.1021/ac5018265. 25157966 10.1021/ac5018265

[32] Andre. Hubaux, Gilbert. Vos, Decision and detection limits for calibration curves. Anal Chem. 1970;42:849–855. 10.1021/ac60290a013.

[33] Matuszewski BK, Constanzer ML, Chavez-Eng CM. Strategies for the assessment of matrix effect in quantitative bioanalytical methods based on HPLC−MS/MS. Anal Chem. 2003;75:3019–30. 10.1021/ac020361s. 12964746 10.1021/ac020361s

[34] Crimmins EM, Zhang YS, Kim JK, Frochen S, Kang H, Shim H, Ailshire J, Potter A, Cofferen J, Faul J. Dried blood spots: effects of less than optimal collection, shipping time, heat, and humidity. Am J Hum Biol. 2020;32: e23390. 10.1002/ajhb.23390. 31922324 10.1002/ajhb.23390 PMC7347424

[35] Yang Y, Jiang J, Jiang Y, Ju Y, He J, Yu K, Kan G, Zhang H. Determination of amino acid metabolic diseases from dried blood spots with a rapid extraction method coupled with nanoelectrospray ionization mass spectrometry. Talanta. 2024;272: 125768. 10.1016/j.talanta.2024.125768. 38340394 10.1016/j.talanta.2024.125768

[36] Alizadeh EA, Rast G, Cantow C, Schiwon J, Krause F, De Meyer GRY, Guns P-J, Guth BD, Markert M. Optimization of bioanalysis of dried blood samples. J Pharmacol Toxicol Methods. 2023;123: 107296. 10.1016/j.vascn.2023.107296. 37482323 10.1016/j.vascn.2023.107296

[37] Griffin EK, Aristizabal-Henao JJ, Bowden JA. Evaluation of different extraction methods for the analysis of per- and polyfluoroalkyl substances in dried blood spots from the Florida manatee (Trichechus manatus). Environ Toxicol Chem. 2021;40:2726–32. 10.1002/etc.5175. 34293220 10.1002/etc.5175

[38] Bojko B. Emerging technologies: analytical lab vs. clinical lab perspective. Common goals and gaps to be filled in the pursuit of green and sustainable solutions. Anal Bioanal Chem. 2024. 10.1007/s00216-024-05139-6. 10.1007/s00216-024-05139-6 38246907

[39] Carignan CC, Bauer RA, Patterson A, Phomsopha T, Redman E, Stapleton HM, Higgins CP. Self-collection blood test for PFASs: comparing volumetric microsamplers with a traditional serum approach. Environ Sci Technol. 2023;57:7950–7. 10.1021/acs.est.2c09852. 37189231 10.1021/acs.est.2c09852 PMC10233751

[40] Lin EZ, Nason SL, Zhong A, Fortner J, Godri Pollitt KJ. Trace analysis of per- and polyfluorinated alkyl substances (PFAS) in dried blood spots – demonstration of reproducibility and comparability to venous blood samples. Sci Total Environ. 2023 883;163530. 10.1016/j.scitotenv.2023.163530. 10.1016/j.scitotenv.2023.163530 PMC10248884 37094673

[41] Gosetti F, Chiuminatto U, Zampieri D, Mazzucco E, Robotti E, Calabrese G, Gennaro MC, Marengo E. Determination of perfluorochemicals in biological, environmental and food samples by an automated on-line solid phase extraction ultra high performance liquid chromatography tandem mass spectrometry method. J Chromatogr A. 2010;1217:7864–72. 10.1016/j.chroma.2010.10.049. 21071035 10.1016/j.chroma.2010.10.049

[42] Hassan M-A, Chen X, Fnu PIJ, Osonga FJ, Sadik OA, Li M, Chen H. Rapid detection of per- and polyfluoroalkyl substances (PFAS) using paper spray-based mass spectrometry. J Hazard Mater. 2024;465: 133366. 10.1016/j.jhazmat.2023.133366. 38185081 10.1016/j.jhazmat.2023.133366

[43] Manousi N, Wojnowski W, Płotka-Wasylka J, Samanidou V. Blue applicability grade index (BAGI) and software: a new tool for the evaluation of method practicality. Green Chem. 2023;25:7598–604. 10.1039/D3GC02347H.

[44] Mahmoud SA, Abbas AEF, katamesh NS. Greenness, whiteness, and blueness assessment with spider chart solvents evaluation of HPTLC-densitometric method for quantifying a triple combination anti-Helicobacter pylori therapy. Sustain Chem Pharm. 2024;37:101412. 10.1016/j.scp.2023.101412.

[45] Jobst KJ, Arora A, Pollitt KG, Sled JG. Dried blood spots for the identification of bioaccumulating organic compounds: current challenges and future perspectives. Environ Toxicol Expo Anal Chall Emerg Expo. 2020;15:66–73. 10.1016/j.coesh.2020.07.001. 10.1016/j.coesh.2020.07.001 PMC7560987 33073071

[46] Baillargeon KR, Mace CR. Microsampling tools for collecting, processing, and storing blood at the point-of-care. Bioeng Transl Med. 2023;8: e10476. 10.1002/btm2.10476. 36925672 10.1002/btm2.10476 PMC10013775

[47] Kotlarz N, McCord J, Collier D, Lea CS, Strynar M, Lindstrom AB, Wilkie AA, Islam JY, Matney K, Tarte P, Polera ME, Burdette K, DeWitt J, May K, Smart RC., Knappe Detlef RU, Hoppin JA. Measurement of novel, drinking water-associated PFAS in blood from adults and children in Wilmington, North Carolina, Environ. Health Perspect. 2020;128:077005. 10.1289/EHP6837. 10.1289/EHP6837 PMC7375159 32697103

[48] Munoz G, Liu J, Vo Duy S, Sauvé S. Analysis of F-53B, Gen-X, ADONA, and emerging fluoroalkylether substances in environmental and biomonitoring samples: a review, Trends Environ. Anal Chem. 2019;23;e00066. 10.1016/j.teac.2019.e00066.

[49] Koponen J, Rudge J, Kushon S, Kiviranta H. Novel volumetric adsorptive microsampling technique for determination of perfluorinated compounds in blood. Anal Biochem. 2018;545:49–53. 10.1016/j.ab.2018.01.015. 29366694 10.1016/j.ab.2018.01.015

[50] Williams ML, Olomukoro AA, Emmons RV, Godage NH, Gionfriddo E. Matrix effects demystified: strategies for resolving challenges in analytical separations of complex samples. J Sep Sci. 2023;46:2300571. 10.1002/jssc.202300571. 10.1002/jssc.202300571 37897324

[51] Gutiérrez‑Martín D, Restrepo‑Montes E, Golovko O, López‑Serna R,· Aalizadeh R, Thomaidis NS, Marquès M, Gago‑Ferrero P, Gil‑Solsona R. Comprehensive profiling and semi‑quantification of exogenous chemicals in human urine using HRMS‑based strategies. Anal Bioanal Chem. 2023;415:7297–7313. 10.1007/s00216-023-04998-9 PMC10684428 37946034

[52] Partington JM, Rana S, Szabo D, Anumol T, Clarke BO. Comparison of high-resolution mass spectrometry acquisition methods for the simultaneous quantification and identification of per- and polyfluoroalkyl substances (PFAS). Anal Bioanal Chem. 2024;416:895–912. 10.1007/s00216-023-05075-x. 38159142 10.1007/s00216-023-05075-x

[53] Stramenga A, Tavoloni T, Stecconi T, Galarini R, Giannotti M, Siracusa M, Ciriaci M, Bacchiocchi S, Piersanti A. Perfluoroalkyl and polyfluoroalkyl substances (PFASs): an optimized LC-MS/MS procedure for feed analysis. J Chromatogr B. 2021;1186: 123009. 10.1016/j.jchromb.2021.123009. 10.1016/j.jchromb.2021.123009 34763303

[54] Poothong S, Papadopoulou E, Lundanes E, Padilla-Sánchez JA, Thomsen C, Haug LS. Dried blood spots for reliable biomonitoring of poly- and perfluoroalkyl substances (PFASs). Sci Total Environ. 2019;655:1420–6. 10.1016/j.scitotenv.2018.11.214. 30577133 10.1016/j.scitotenv.2018.11.214

[55] Koelmel JP, Lin EZ, Parry E, Stelben P, Rennie EE, Godri Pollitt KJ. Novel perfluoroalkyl substances (PFAS) discovered in whole blood using automated non-targeted analysis of dried blood spots. Sci Total Environ. 2023;883:163579. 10.1016/j.scitotenv.2023.163579. 10.1016/j.scitotenv.2023.163579 PMC10247435 37100129

