# Supplementary material for: Development and validation of the UHPLC-MS/MS method for the quantitative determination of 25 PFAS in dried blood spots
Source: Anal Bioanal Chem. 2024 Aug 19;416(26):5671–87. doi: 10.1007/s00216-024-05484-6 (PMC11493788; doi:10.1007/s00216-024-05484-6)
Supplement: Supplementary file 1 — Supplementary file1 (DOCX 36 KB) [file 216_2024_5484_MOESM1_ESM.docx]

Table S1. Intra- and inter-day accuracy and precision are reported as bias% and CV% at each concentration level of the calibration curve and as averaged values for each target analyte.

| **Analyte** | **Concentration (ng/ml)** | **Accuracy intra-day**  **(bias ± %)** | **Accuracy inter-day**  **(bias ± %)** | **Precision intra-day**  **(cv %)** | **Precision**  **Inter-day**  **(cv %)** |
| --- | --- | --- | --- | --- | --- |
| **Perfluorocarboxylic acids** |  |  |  |  |  |
| PFBA | 2  5  10  20  50  100  **Average** | 19%  7%  -11%  -18%  -16%  -19%  **-6%** | 19%  3%  -16%  -20%  -16%  -8%  **-6%** | 5%  4%  4%  4%  6%  12%  **6%** | 5%  11%  6%  4%  7%  10%  **7%** |
| PFPeA | 2  5  10  20  50  100  **Average** | 17%  -10%  -8%  -1%  -8%  -16%  **-4%** | 17%  -11%  -8%  -1%  -8%  12%  **1%** | 5%  2%  5%  10%  9%  9%  **7%** | 6%  3%  10%  10%  11%  14%  **9%** |
| PFHxA | 2  5  10  20  50  100  **Average** | 20%  3%  -17%  -20%  -19%  -13%  **-8%** | 18%  15%  -10%  -18%  -20%  -15%  **-5%** | 4%  4%  5%  6%  7%  17%  **7%** | 8%  14%  7%  9%  13%  20%  **12%** |
| PFHpA | 2  5  10  20  50  100  **Average** | 19%  -1%  -14%  -17%  -15%  -6%  **-6%** | 20%  1%  -13%  -16%  -13%  16%  **-1%** | 6%  6%  4%  4%  7%  13%  **7%** | 6%  5%  3%  3%  12%  16%  **8%** |
| PFOA | 2  5  10  20  50  100  **Average** | 10%  -13%  -18%  -20%  -19%  -17%  **-13%** | 15%  8%  -18%  -9%  -19%  -17%  **-7%** | 5%  3%  8%  8%  7%  8%  **7%** | 6%  5%  10%  8%  10%  15%  **9%** |
| PFNA | 2  5  10  20  50  100  **Average** | 16%  -1%  -18%  -16%  -17%  -4%  **-7%** | 17%  11%  -13%  -16%  -10%  -5%  **-3%** | 10%  7%  10%  13%  14%  13%  **11%** | 11%  8%  11%  14%  14%  18%  **13%** |
| PFDA | 2  5  10  20  50  100  **Average** | 11%  4%  -8%  -8%  -4%  -18%  **-4%** | 10%  8%  -9%  -8%  -5%  6%  **0.3%** | 9%  8%  9%  7%  8%  10%  **9%** | 11%  11%  12%  9%  10%  11%  **11%** |
| PFUdA | 2  5  10  20  50  100  **Average** | 7%  4%  7%  5%  8%  11%  **7%** | 8%  8%  12%  12%  15%  16%  **12%** | 1%  9%  13%  6%  14%  7%  **8%** | 7%  1%  6%  2%  3%  9%  **5%** |
| PFDoA | 2  5  10  20  50  100  **Average** | 12%  2%  -10%  -8%  -7%  -8%  **-3%** | 17%  16%  -3%  -7%  -10%  6%  **3%** | 4%  6%  5%  8%  5%  8%  **6%** | 5%  6%  5%  7%  6%  8%  **6%** |
| PFTrDA | 2  5  10  20  50  100  **Average** | 10%  2%  -7%  -5%  -3%  -20%  **-4%** | 10%  1%  -7%  -6%  -3%  6%  **0.2%** | 7%  11%  7%  9%  10%  7%  **9%** | 9%  12%  8%  9%  10%  9%  **10%** |
| PFTeDA | 2  5  10  20  50  100  **Average** | 7%  -2%  1%  -3%  -2%  -19%  **-3%** | 6%  -2%  -1%  -3%  -3%  3%  **0%** | 15%  14%  9%  13%  13%  14%  **13%** | 14%  20%  12%  12%  14%  14%  **14%** |
| PFHxDA | 2  5  10  20  50  100  **Average** | 9%  -19%  6%  1%  -2%  3%  **-0.3%** | 9%  -18%  6%  1%  -1%  2%  **-0.2%** | 1%  12%  6%  4%  12%  10%  **8%** | 1%  15%  6%  5%  18%  13%  **10%** |
| PFODA | 2  5  10  20  50  100  **Average** | -3%  4%  -5%  2%  3%  -1%  **0%** | -3%  4%  -5%  2%  4%  -2%  **0%** | 5%  6%  4%  3%  8%  7%  **6%** | 4%  10%  8%  4%  17%  7%  **8%** |
| **Perfluorosulfonic acids** |  |  |  |  |  |
| L-PFBS | 2  5  10  20  50  100  **Average** | -5%  -1%  6%  1%  -1%  -19%  **-3%** | -5%  -1%  5%  2%  -2%  2%  **0.2%** | 4%  4%  4%  10%  11%  10%  **7%** | 5%  10%  6%  16%  14%  12%  **11%** |
| L-PFHxS | 2  5  10  20  50  100  **Average** | 4%  2%  -4%  -2%  -2%  1%  **-0.2%** | 3%  2%  -4%  -2%  -1%  2%  **0%** | 3%  6%  9%  8%  9%  1%  **6%** | 3%  7%  9%  9%  10%  1%  **7%** |
| L-PFOS | 2  5  10  20  50  100  **Average** | 5%  -1%  -16%  -13%  -16%  -16%  **-10%** | 19%  11%  -5%  -4%  -11%  12%  **4%** | 1%  5%  2%  4%  2%  4%  **3%** | 3%  10%  3%  4%  5%  12%  **6%** |
| L-PFDS | 2  5  10  20  50  100  **Average** | 1%  4%  1%  -3%  -3%  -2%  **-0.3%** | 4%  6%  1%  -3%  -2%  -2%  **1%** | 11%  6%  7%  8%  7%  7%  **8%** | 11%  15%  14%  11%  10%  7%  **11%** |
| **Replacement PFAS** |  |  |  |  |  |
| HFPO-DA | 2  5  10  20  50  100  **Average** | -2%  3%  -5%  5%  2%  -1%  **0.3%** | -1%  3%  -5%  5%  2%  -1%  **1%** | 9%  12%  12%  8%  12%  8%  **10%** | 17%  14%  14%  13%  14%  15%  **15%** |
| ADONA | 2  5  10  20  50  100  **Average** | -12%  -4%  4%  8%  5%  -2%  **-0.2%** | -12%  -4%  5%  8%  5%  -1%  **0.2%** | 1%  6%  13%  15%  12%  13%  **10%** | 6%  6%  18%  19%  14%  19%  **14%** |
| 9Cl-PF3ONS | 2  5  10  20  50  100  **Average** | 18%  -6%  -12%  -20%  -10%  -17%  **-8%** | 18%  -3%  -9%  -16%  -7%  -13%  **-5%** | 3%  12%  12%  5%  12%  7%  **9%** | 3%  13%  15%  4%  18%  17%  **12%** |
| 11Cl-PF3OUdS | 2  5  10  20  50  100  **Average** | 14%  -6%  -14%  -14%  -13%  4%  **-5%** | 16%  7%  -5%  -8%  -10%  7%  **1%** | 5%  9%  11%  11%  7%  7%  **8%** | 5%  13%  15%  15%  12%  14%  **12%** |
| **Perfluoroalkylethers** |  |  |  |  |  |
| PF4OPeA | 2  5  10  20  50  100  **Average** | 1%  -1%  -1%  4%  -3%  1%  **0.2%** | 1%  -1%  -1%  4%  -3%  1%  **0.2%** | 9%  5%  10%  6%  10%  6%  **7%** | 9%  8%  11%  9%  9%  6%  **9%** |
| PF5OHxA | 2  5  10  20  50  100  **Average** | -17%  -1%  14%  3%  -1%  -19%  **-4%** | -16%  -1%  14%  2%  -4%  1%  **-1%** | 12%  7%  6%  11%  7%  8%  **9%** | 10%  7%  9%  13%  8%  9%  **9%** |
| PFEESA | 2  5  10  20  50  100  **Average** | 6%  1%  -3%  -4%  -2%  -19%  **-4%** | 8%  1%  -2%  -3%  -2%  4%  **1%** | 5%  7%  11%  12%  5%  7%  **8%** | 12%  12%  15%  16%  9%  14%  **13%** |
| 3,6-OPFHpA | 2  5  10  20  50  100  **Average** | -16%  16%  9%  4%  3%  -1%  **3%** | -12%  11%  2%  -3%  1%  2%  **0.2%** | 10%  11%  17%  16%  11%  12%  **13%** | 10%  10%  15%  18%  14%  13%  **12%** |

Table S2. Quantitative PFASs results obtained by spiking the *Capitainer*®*B* cards with either whole blood (WB-S) or plasma (P-S) at the final concentration of 15 ng/mL and 30 ng/mL against calibration curves built on blank whole blood (DBS-cal) or blank plasma (DPS-cal).

| **Analyte** | **15 ng/mL WB-S** | **15 ng/mL P-S** | **30 ng/mL WB-S** | **30 ng/mL P-S** |
| --- | --- | --- | --- | --- |
| PFBA DBS-cal | 15.21 | 15.60 | 31.36 | 29.69 |
| PFBA DPS-cal | 15.21 | 15.67 | 31.31 | 29.26 |
| PFPeA DBS-cal | 15.61 | 14.38 | 30.87 | 27.28 |
| PFPeA DPS-cal | 15.57 | 14.52 | 31.61 | 27.02 |
| PFHxA DBS-cal | 14.92 | 14.32 | 31.70 | 27.20 |
| PFHxA DPS-cal | 15.82 | 17.15 | 34.87 | 28.78 |
| PFHpA DBS-cal | 14.25 | 15.43 | 30.88 | 28.07 |
| PFHpA DPS-cal | 15.15 | 16.48 | 32.31 | 30.80 |
| PFOA DBS-cal | 16.75 | 15.38 | 29.09 | 29.72 |
| PFOA DPS-cal | 16.36 | 16.66 | 28.42 | 29.98 |
| PFNA DBS-cal | 14.16 | 14.12 | 31.59 | 27.54 |
| PFNA DPS-cal | 14.90 | 16.87 | 33.41 | 32.09 |
| PFDA DBS-cal | 14.28 | 14.76 | 28.01 | 30.61 |
| PFDA DPS-cal | 14.99 | 15.49 | 30.39 | 27.96 |
| PFUdA DBS-cal | 16.73 | 14.35 | 31.14 | 33.33 |
| PFUdA DPS-cal | 15.98 | 14.68 | 29.47 | 32.05 |
| PFDoA DBS-cal | 14.38 | 15.06 | 29.45 | 32.55 |
| PFDoA DPS-cal | 14.90 | 14.35 | 30.50 | 30.62 |
| PFTrDA DBS-cal | 14.26 | **9.43^*^** | 32.54 | **22.48^*^** |
| PFTrDA DPS-cal | 14.68 | 14.34 | 33.50 | 29.43 |
| PFTeDA DBS-cal | 15.06 | **9.07^*^** | 32.81 | **20.94^*^** |
| PFTeDA DPS-cal | 17.13 | 14.41 | 32.74 | 30.95 |
| PFHxDA DBS-cal | 14.22 | **9.36^*^** | 28.79 | **16.99^*^** |
| PFHxDA DPS-cal | 18.44 | 13.94 | 33.47 | 28.04 |
| PFODA DBS-cal | 16.95 | **9.55^*^** | 27.68 | **17.97^*^** |
| PFODA DPS-cal | 15.92 | 14.54 | 27.40 | 27.13 |
| L-PFBS DBS-cal | 13.91 | 13.76 | 31.68 | 27.41 |
| L-PFBS DPS-cal | 16.30 | 15.40 | 34.05 | 27.19 |
| L-PFHxS DBS-cal | 15.21 | 17.02 | 33.54 | 30.02 |
| L-PFHxS DPS-cal | 14.76 | 15.34 | 32.65 | 27.14 |
| L-PFOS DBS-cal | 16.03 | 15.61 | 28.26 | 28.34 |
| L-PFOS DPS-cal | 15.39 | 14.98 | 27.95 | 27.51 |
| L-PFDS DBS-cal | 15.11 | 15.61 | 32.92 | 28.88 |
| L-PFDS DPS-cal | 16.49 | 14.98 | **47.13^*^** | 28.30 |
| HFPO-DA DBS-cal | 15.87 | 16.26 | 28.91 | 29.44 |
| HFPO-DA DPS-cal | 15.35 | 17.93 | 27.97 | 28.48 |
| ADONA DBS-cal | 15.41 | 13.86 | 28.83 | 28.18 |
| ADONA DPS-cal | 15.36 | 16.06 | 33.63 | 33.86 |
| 9ClPF3ONS DBS-cal | 16.18 | 16.86 | 33.34 | 33.99 |
| 9ClPF3ONS DPS-cal | 14.60 | 13.55 | 27.52 | 27.35 |
| 11ClPF3OUdS DBS-cal | 15.19 | 17.17 | 32.42 | 32.18 |
| 11ClPF3OUdS DPS-cal | 14.91 | 15.42 | 31.80 | 27.56 |
| PF4OPeA DBS-cal | 14.06 | 13.34 | 29.76 | 28.14 |
| PF4OPeA DPS-cal | 14.75 | 13.41 | 32.23 | 30.42 |
| PF5OHxA DBS-cal | 15.00 | 13.45 | 29.40 | 31.15 |
| PF5OHxA DPS-cal | 16.24 | 15.26 | 31.82 | 32.18 |
| PFEESA DBS-cal | 14.46 | 13.98 | 32.44 | 31.13 |
| PFEESA DPS-cal | 16.04 | 14.18 | 32.98 | 31.41 |
| 3,6-OPFHpA DBS-cal | 14.74 | 15.83 | 31.87 | 29.97 |
| 3,6-OPFHpA DPS-cal | 15.99 | 17.19 | 32.71 | 32.60 |
